# Bojungikki-Tang Augments Pembrolizumab Efficacy in Human PBMC-Injected H460 Tumor-Bearing Mice

**DOI:** 10.3390/life14101246

**Published:** 2024-09-30

**Authors:** Se Won Na, Jin-Mu Yi, Heerim Yeo, Sang-Min Park, Mibae Jeong, Jaemoo Chun, Mi-Kyung Jeong

**Affiliations:** 1 KM Convergence Research Division, Korea Institute of Oriental Medicine, Daejeon 34054, Republic of Korea; sewon3066@kiom.re.kr (S.W.N.); jmyi@kiom.re.kr (J.-M.Y.); mebelly@kiom.re.kr (M.J.); 2 College of Pharmacy, Chungnam National University, Daejeon 34134, Republic of Korea; 202350584@o.cnu.ac.kr (H.Y.); smpark@cnu.ac.kr (S.-M.P.)

**Keywords:** Bojungikki-Tang, non-small cell lung cancer, anti-tumor, immunoregulation, humanized mouse model

## Abstract

Bojungikki-Tang (BJIKT) is traditionally used to enhance digestive function and immunity. It has gained attention as a supplement to chemotherapy or targeted therapy owing to its immune-boosting properties. This study aimed to evaluate the synergistic anti-tumor effects of BJIKT in combination with pembrolizumab in a preclinical model. MHC I/II double knockout NSG mice were humanized with peripheral blood mononuclear cells (PBMCs) and injected subcutaneously with H460 lung tumor cells to establish a humanized tumor model. Both agents were administered to evaluate their impact on tumor growth and immune cell behavior. Immunohistochemistry showed decreased exhaustion markers in CD8(+) and CD4(+) T cells within the tumor, indicating enhanced T cell activity. Additionally, RNA sequencing, transcriptome analysis, and quantitative PCR analysis were performed on tumor tissues to investigate the molecular mechanisms underlying the observed effects. The results confirmed that BJIKT improved T cell function and tumor necrosis factor signaling while suppressing transforming growth factor-β signaling. This modulation led to cell cycle arrest and apoptosis. These findings demonstrate that BJIKT, when combined with pembrolizumab, produces significant anti-tumor effects by altering immune pathways and enhancing the anti-tumor immune response. This study provides valuable insights into the role of BJIKT in the tumor microenvironment and its potential to improve therapeutic outcomes.

## 1. Introduction

Non-small-cell lung cancer (NSCLC) is the predominant form of lung cancer, comprising approximately 85% of all cases diagnosed across the disease spectrum [1,2]. This remains a formidable challenge in oncology, necessitating innovative approaches to enhance treatment outcomes [3]. Recent years have seen the emergence of immune checkpoint inhibitors (ICIs) as a primary therapeutic approach, which has brought new hope to the field [4]. However, their efficacy remains limited, with only a subset of patients demonstrating a positive response [5]. Consequently, ongoing research is actively exploring strategies to overcome resistance to ICIs, including combination therapies, biomarkers for patient selection, and novel agents that modulate the tumor microenvironment, to improve the therapeutic landscape for NSCLC [6]. To overcome this limitation, there is growing interest in investigating combination therapies involving complementary and alternative medicines, such as herbal remedies, to augment the efficacy of ICIs.

Bojungikki-Tang (BJIKT), derived from a blend of 10 therapeutic herbs, is extensively utilized to treat digestive disorders and enhance appetite [7]. It is widely employed in cancer therapy and as an adjunctive treatment, particularly for addressing cancer-related fatigue. It has garnered attention for its potential synergistic effects when combined with existing anticancer therapies [8,9,10,11,12]. Additionally, BJIKT plays a role in alleviating and managing chemotherapy-induced leukopenia and exhibits significant immunomodulatory properties when paired with immunotherapy in models of NSCLC [13,14,15]. This study explored the potential synergies between BJIKT and pembrolizumab, a humanized monoclonal anti-PD-1 antibody, for treating NSCLC.

The primary objective of this study was to examine the immune-mediated anti-tumor effects resulting from the co-administration of BJIKT and pembrolizumab in a preclinical model of NSCLC. Humanized mouse models were selected for this study because of their capacity to replicate the human tumor microenvironment (TME) more accurately than alternative models, such as the KLN205 tumor syngeneic model, which presents challenges due to microenvironmental discrepancies. We used a MHC I/II double knockout (dKO) NSG mouse model, implanted with H460 human lung cancer cells and injected with human peripheral blood mononuclear cells (PBMCs). This model was chosen because it allows for a more precise evaluation of immune interactions in a system where key components of the human immune response are present, yet the absence of MHC I/II molecules minimizes the potential for direct immune recognition by the host’s own immune system [16,17]. The combined administration model of BJIKT and pembrolizumab enables a comprehensive assessment of the immune response and tumor growth.

Specifically, we investigated the effect of combined BJIKT and pembrolizumab therapy on tumor growth, characterized alterations in immune cell populations within the TME, and assessed changes in gene expression profiles related to immune responses and anti-tumor effects. By addressing these objectives, we aimed to reveal the potential of BJIKT to modulate the immune environment in NSCLC and enhance the therapeutic efficacy of immunotherapy.

## 2. Materials and Methods

### 2.1. Animals

The MHC I/II dKO NSG mouse model, humanized with pre-characterized PBMC donors for human immune cell engraftment, was purchased from Jackson Laboratory (Bar Harbor, ME, USA). H460 cells (1 × 10^6^ cells), a human NSCLC cell line acquired from the American Type Culture Collection (Manassas, VA, USA), were subcutaneously injected into human PBMC-injected MHC I/II dKO NSG mice to establish a tumor model. Five days post-injection, mice were randomly divided into four distinct groups with similar average tumor sizes when the tumor reached an average size of 50 mm^3^: control, pembrolizumab, BJIKT, and combination (BJIKT and pembrolizumab). The mice were administered a daily oral dose of 450 mg/kg BJIKT or received 10 mg/kg pembrolizumab via intraperitoneal injection every three days. The experimental protocols were approved by the Animal Care and Use Committee of the Korea Institute of Oriental Medicine (Approval No. 21-085).

### 2.2. Chemicals and Reagents

BJIKT extract was obtained from Hanpoong Pharmaceutical Co. (Jeonju, Republic of Korea), and pembrolizumab (anti-PD-1 antibody) was purchased from MSD Korea Ltd. (Seoul, Republic of Korea). BJIKT, a water-based extract, comprises 10 herbs: *Astragalus membranaceus* (Fisch.) Bunge (16.6%), *Atractylodes macrocephala* Koidz. (16.6%), *Panax ginseng* C. A. Mey. (16.6%), *Angelica gigas* Nakai (12.5%), *Ziziphus jujuba* var. inermis (Bunge) Rehder (8.4%), *Bupleurum falcatum* L. (8.4%), *Citrus unshiu* Marcow. (8.4%), *Glycyrrhiza glabra* L. (6.3%), *Cimicifuga heracleifolia* Kom. (4.1%), and *Zingiber officinale* Rocoe (2.1%). BJIKT was prepared as described previously [14]. The herbs were subjected to aqueous extraction at 95 °C, followed by filtration, evaporation under reduced pressure, and lyophilization.

### 2.3. Immunohistochemistry (IHC) and Terminal Deoxynucleotidyl Transferase dUTP Nick End Labeling (TUNEL) Staining

For IHC staining, paraffin-embedded tumor tissue sections underwent a 10-min treatment with 3% H_2_O_2_ after prior boiling in Tris-EDTA antigen retrieval solution. Super Block (ScyTek Laboratories, Logan, UT, USA) was utilized for blocking, succeeded by overnight incubation at 4 °C with primary antibodies, such as Ki-67 (ab15580, Abcam, Cambridge, UK), CD3 (MBS462027, MyBioSource, San Diego, CA, USA), CD4 (MAB15251, Abnova, Taipei City, Taiwan), CD8 (ab237709, Abcam), lymphocyte activation gene-3 (LAG-3) (ab209236, Abcam), T cell immunoreceptor with immunoglobulin and ITIM domain (TIGIT) (ab243903, Abcam), and T cell immunoglobulin and mucin domain-containing-3 (TIM-3) (ab241332, Abcam). Subsequently, a secondary antibody reaction was performed at room temperature using a DAB Detection System (Vector Laboratories, Newark, CA, USA). The sections were mounted on gelatin-coated slides, dehydrated, and secured on coverslips.

For TUNEL staining, paraffin-embedded tumor tissue sections were processed using the DeadEnd™ Colorimetric TUNEL System (Promega, Madison, WI, USA). Briefly, tissue sections were deparaffinized in xylene and rehydrated using a graded ethanol series. Tissue sections were fixed in 4% paraformaldehyde for 15 min, followed by permeabilization with 20 μg/mL Proteinase K for 10–30 min at room temperature. After additional fixation, sections were equilibrated with Equilibration Buffer and labeled with TdT reaction mix for 60 min at 37 °C. The reaction was stopped, and endogenous peroxidase activity was blocked using 0.3% hydrogen peroxide. Streptavidin-HRP (1:500) was applied, followed by DAB staining. Finally, slides were washed, mounted, and visualized under a light microscope.

### 2.4. RNA Isolation for the RNA-seq Data

Total RNA was extracted from H460 tumor tissues in mice treated with vehicle, pembrolizumab, and/or BJIKT using TRIzol reagent (Invitrogen, Carlsbad, CA, USA), following the manufacturer’s protocol. For sequencing library construction, 500 ng of extracted RNA was used. RNA integrity was evaluated using the Agilent TapeStation 4000 system (Agilent Technologies, Amstelveen, The Netherlands), and RNA concentration was determined using a ND-2000 Spectrophotometer (Thermo Fischer Scientific Inc., Wilmington, DE, USA).

### 2.5. Library Preparation and Sequencing of the RNA-seq Data

Library construction was performed using the QuantSeq 3 mRNA-Seq Library Prep Kit (Lexogen, Inc., Vienna, Austria) following the provided guidelines. In summary, total RNA was prepared and hybridized with an oligo-dT primer containing an Illumina-compatible sequence, followed by reverse transcription. RNA template degradation preceded the initiation of second-strand synthesis by using a random primer with an Illumina-compatible linker. After purifying the double-stranded library to eliminate reaction components, PCR amplification introduced the necessary adapter sequences for cluster generation, with a final library purification from the PCR materials. High-throughput sequencing was performed as single-end 75 sequencing using a NextSeq 550 (Illumina, Inc., San Diego, CA, USA).

### 2.6. Transcriptome Analysis of the RNA-seq Data

Transcriptome analysis of the RNA-seq data was performed using R software (v4.1.3). The DESeq2 package (v1.38.3) was utilized for differential expression analysis. This analysis identified significant differentially expressed genes (DEGs), applying criteria of an adjusted *p*-value of 0.05 and a fold change of 1.5. The identified DEGs were visualized using a volcano plot created with the ggplot2 package (v3.4.4). Gene Set Enrichment Analysis (GSEA) was performed using the fgsea package (v1.28.0) on gene lists ranked based on the results of the differential expression analysis, with the parameters set to minSize = 15, maxSize = 500, and nperm = 10,000. The gene sets used were curated from the Molecular Signatures Database via the msigdbr package (v7.5.1). GSEA calculated the normalized enrichment scores and *p*-values to identify significantly enriched functional terms, indicative of the strength and relevance of gene set associations. The drug–pathway–gene network, which included DEGs in key pathways identified through GSEA, was visualized using Cytoscape software (v3.10.1; Institute for Systems Biology, Seattle, WA, USA).

### 2.7. Cell Culture

T cells were enriched from human PBMCs (Lonza, Morrisville, NC, USA) and expanded in T cell expansion medium (STEMCELL Technologies, Vancouver, BC, Canada) using a human CD3/CD28/CD2 T cell activator (STEMCELL Technologies) with the addition of 100 U/mL recombinant human interleukin (IL)-2 (PeproTech, Rocky Hill, NJ, USA) for 48 h. H460 cells were cultured in RPMI-1640 medium supplemented with 10% FBS, 100 U/mL penicillin, and 100 μg/mL streptomycin at 37 °C in a humidified atmosphere containing 5% CO_2_. Cells were passaged every 2–3 days to maintain exponential growth and used between passages 5 and 20 for all experiments.

### 2.8. Quantitative PCR

Total RNA was extracted from the cells using an RNA extraction kit (RNeasy Plus Mini Kit, Qiagen, Valencia, CA, USA). The concentration and purity of RNA were determined using a Nanodrop (Thermo Fischer Scientific Inc.). cDNA was synthesized from the isolated RNA using a reverse transcription kit (iScript^TM^ Advanced cDNA Synthesis Kit for RT-qPCR, Bio-Rad, Hercules, CA, USA). PCR amplification was performed using specific primers designed for target genes. The primer sequences used were as follows: SPTAN1 (forward 5′-CTG AAG GTC TCA TGG CAG AGG A-3′, reverse 5′-CAC GGT GTG AAC CAT CAG ACG A-3′); ACIN1 (forward 5′-CTC GTT CAG CAT CAA GCA ACA GC-3′, reverse 5′-TTG TGG CAC TGG TGG AGT TGC A-3′); SLC11A1 (forward 5′-CAT CCT CAC GTT CAC CAG CAT G-3′, reverse 5′-CCA CGA AGT AGA GGT TGA TGG C-3′); CR1 (forward 5′-TAG GTG TCA GCC TGG CTT TGT C-3′, reverse 5′-GAC ATC TGG AGG TGG CTG ACA T-3′); FOXP3 (forward 5′-GGC ACA ATG TCT CCT CCA GAG A-3′, reverse 5′-CAG ATG AAG CCT TGG TCA GTG C-3′); PIK3CD (forward 5′-TGC CAA ACC ACC TCC CAT TCC T-3′, reverse 5′-CAT CTC GTT GCC GTG GAA AAG C-3′); PLCG1 (forward 5′-CAT CAC GCA CTA CCA GCA GGT G-3′, reverse 5′-GAC GCG CAT TAG CAT GTG CTC A-3′); BMI1 (forward 5′-GGT ACT TCA TTG ATG CCA CAA CC-3′, reverse 5′-CTG GTC TTG TGA ACT TGG ACA TC-3′); LYRM7 (forward 5′-ATG ATG CCA GAG CAT TAG AAG CAG -3′, reverse 5′-ACC TTG TAT AAC AGA TGT TCT GAG T-3′); CSNK1G1 (forward 5′-GGA GGA CTT GTT TGA CCT CTG TG-3′, reverse 5′-GAC CAA TCA GGA AGT TCT CTG GC-3′); NMI (forward 5′-GAA ACG GAG TTA CAA GAG GCT AC-3′, reverse 5′-GAC AAC TGG CTG TCA TTC TCA GG-3′); UCHL3 (forward 5′-CAA ACA ATC AGC AAT GCC TGT GG-3′, reverse 5′-GGC TCA TTG ACA CAG ATT CCT CC-3′); MTMR4 (forward 5′-CTG TGT TCC TCC AGT GGC TTG A-3′, reverse 5′-TGC CGT AGA GGC AGG AGT ATG T-3′); RPS27A (forward 5′-GCA GAG ACT GAT CTT TGC TGG C-3′, reverse 5′-CTT GGG AGT GGT GTA AGA CTT CT-3′). Gene expression analysis was conducted using the SsoAdvanced SYBR Green Supermix kit (Bio-Rad) on a Bio-Rad CFX Connect Real-Time System (Bio-Rad). The amplification conditions were set as follows: initial denaturation at 95 °C for 2 min, followed by 40 cycles of amplification at 95 °C for 5 s and 60 °C for 30 s.

### 2.9. Statistical Analysis

All statistical analyses were performed using GraphPad Prism 9 (GraphPad Software, San Diego, CA, USA). Data are presented as the mean ± standard deviation (SD). Statistical comparisons between different groups were performed using a two-tailed Student’s *t*-test or ANOVA, followed by Tukey’s post hoc test for multiple comparisons. Statistical significance was set at *p* < 0.05.

## 3. Results

### 3.1. Combination of BJIKT and Pembrolizumab Inhibited Tumor Growth in Human PBMC-Injected H460 Tumor-Bearing MHC I/II dKO NSG Mice

**Figure 1:**
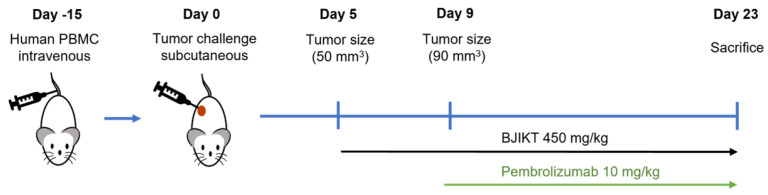
Experimental scheme with a timeline of the mouse experiment. MHC I/II dKO NSG mice humanized with pre-characterized PBMC donors were subcutaneously injected with the human NSCLC cell line H460 (1 × 10^6^ cells). After five days, the mice were segregated into four distinct groups: control, pembrolizumab, BJIKT, and a combination group receiving both BJIKT and pembrolizumab. Treatment consisted of a daily oral administration of 450 mg/kg BJIKT and intraperitoneal injection of 10 mg/kg pembrolizumab every three days.

To understand the progression of tumor growth, changes in tumor volume were monitored throughout the experimental period by measuring them twice weekly (Figure 1).

**Figure 2:**
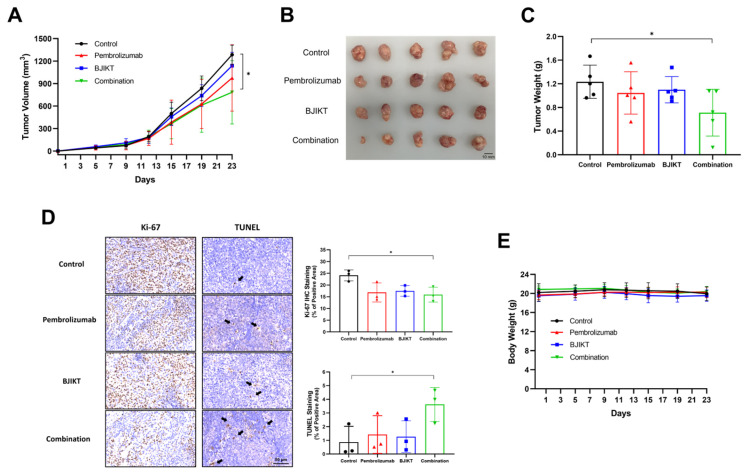
The combination of BJIKT and pembrolizumab inhibited tumor growth in human PBMC-injected H460 tumor-bearing MHC I/II dKO NSG mice. (**A**) Tumor volume changes in human PBMC-injected H460 tumor-bearing MHC I/II dKO NSG mice. The tumor sizes were measured twice weekly. (**B**) Tumor image from each group (*n* = 5 per group). (**C**) Tumor weight changes. (**D**) Representative images of Ki-67 IHC staining and TUNEL staining analysis, and quantitative analysis of both staining (*n* = 3 per group). Black arrows indicate the marked cells in each image. (**E**) Body weight changes in human PBMC-injected H460 tumor-bearing MHC I/II dKO NSG mice. Values are presented as the mean ± SD (*n* = 5 per group). * *p* < 0.05 compared to the combination group.

The results revealed a significant reduction in tumor volume in the group treated with the combination of BJIKT and pembrolizumab relative to the control group (Figure 2A). Tumors in the group treated with BJIKT and pembrolizumab appeared smaller and exhibited reduced growth compared to those in the other groups (Figure 2B). Furthermore, a notable decrease in tumor weight was observed in the group receiving combination therapy, underscoring its efficacy in impeding tumor growth compared to the control group (Figure 2C). Additionally, we confirmed that the expression of Ki-67 was significantly reduced in the group treated with the combination of BJIKT and pembrolizumab, while TUNEL staining showed a tendency to increase, indicating enhanced apoptosis (Figure 2D). Furthermore, no significant variation in body weight was detected across the groups (Figure 2E). These results suggest that the combined treatment did not induce significant changes in body weight, indicating the absence of apparent toxicity affecting body weight.

### 3.2. BJIKT and Pembrolizumab Combination Enhanced Immune Cell Infiltration and Reduced T Cell Exhaustion Markers

**Figure 3:**
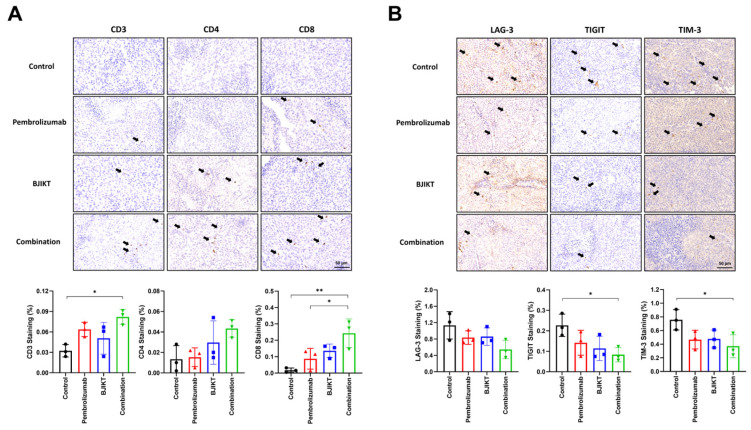
The combination of BJIKT and pembrolizumab increased immune cell infiltration in tumors and decreased the exhaustion markers of T cells. (**A**) IHC staining for CD3, CD4, and CD8 antibodies in tumor tissues. (**B**) IHC staining using LAG-3, TIGIT, and TIM-3 antibodies in tumor tissues. Black arrows indicate the marked cells in each image. Representative stained sections are shown (scale bar, 50 μm). Values are presented as the mean ± SD (*n* = 3 per group). * *p* < 0.05 and ** *p* < 0.01 compared between each group.

IHC staining using antibodies against CD3, CD4, and CD8 was performed on tumor tissues to assess immune cell infiltration. The results demonstrated a significant increase in the infiltration of CD3(+) T cells in tumors treated with the combination of BJIKT and pembrolizumab compared to the control group, with CD4(+) T cells also showing a trend toward an increase, though not statistically significant. Specifically, the IHC staining of CD8(+) T cells in the tumor tissue showed that, while there was no significant increase in CD8(+) T cell expression in the BJIKT monotherapy or pembrolizumab monotherapy groups compared to the control group, a significant increase was observed in the combination group. Notably, the expression of CD8(+) T cells was higher in the combination group compared to the pembrolizumab monotherapy group (Figure 3A). The presence of these enhanced immune cells in the TME suggests an anti-tumor immune response induced by the combination treatment. In addition, IHC staining for exhaustion markers, including LAG-3, TIGIT, and TIM-3, was conducted on the tumor tissues to evaluate their impact on T cell exhaustion (Figure 3B). Combination treatment led to a marked reduction in the expression of these exhaustion markers relative to the control groups. This reduction in exhaustion markers implies the mitigation of T cell exhaustion. These findings indicate that combination therapy with BJIKT and pembrolizumab in the TME specifically focuses on immune cell infiltration and the expression of exhaustion markers on T cells in human PBMC-injected H460 tumor-bearing MHC I/II dKO NSG mice.

### 3.3. Transcriptome Analysis Revealed the Target Signaling Pathways of BJIKT in NSCLC Treatment

**Figure 4:**
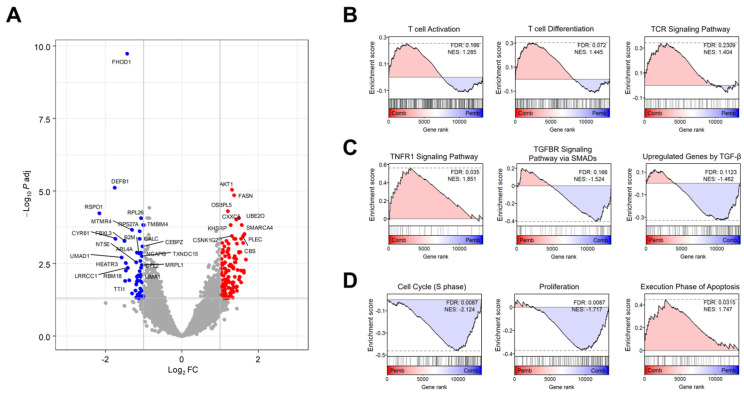
Transcriptome analysis of the combination therapy involving BJIKT and pembrolizumab. (**A**) A Volcano plot illustrating the variance in gene expression between combination therapy (Comb) involving BJIKT and pembrolizumab (Pemb), in contrast to a single treatment with pembrolizumab. Significantly upregulated and downregulated differentially expressed genes are represented as red and blue dots, respectively. (**B**–**D**) GSEA results comparing combination therapy involving BJIKT and pembrolizumab to a single treatment with pembrolizumab. GSEA plots using gene sets associated with (**B**) the functions of the T cells (“T cell activation”, “T cell differentiation”, and “TCR signaling pathway”); (**C**) TNF and TGF-β signaling (“TNFR1 signaling pathway”, “TGFBR signaling pathway via SMADS”, and “upregulated genes by TGF-β”); and (**D**) cell phenotypes (“cell cycle, S phase”, “proliferation”, and “execution phase of apoptosis”). FDR, false discovery rate; NES, normalized enrichment score; Padj, adjusted *p*-value.

To investigate the combined effects of BJIKT and pembrolizumab, we performed a comprehensive transcriptome analysis using RNA-seq data from human PBMC-injected H460 tumor-bearing MHC I/II dKO NSG mice treated with pembrolizumab and/or BJIKT. Differential expression analysis identified a distinct gene expression profile for the combined therapy compared to pembrolizumab alone, including 554 DEGs, with 301 upregulated and 253 downregulated genes (Figure 4A). GSEA was performed to delineate the synergistic effects of the BJIKT and pembrolizumab combination at the cellular pathway level (Figure 4B–D). Combination treatment with BJIKT modulated the function of T cells by increasing the expression of genes associated with T cell activation, differentiation, and the TCR signaling pathway (Figure 4B). This treatment also activated the immunity-promoting tumor necrosis factor (TNF) signaling pathway and suppressed the immunosuppressive transforming growth factor-β (TGF-β) signaling pathway (Figure 4C). Regarding cellular phenotype markers, the pathways associated with cell cycle and proliferation were suppressed. Conversely, those associated with apoptosis were enhanced following treatment with BJIKT (Figure 4D).

### 3.4. Network Analysis Regarding the Molecular Target of BJIKT

**Figure 5:**
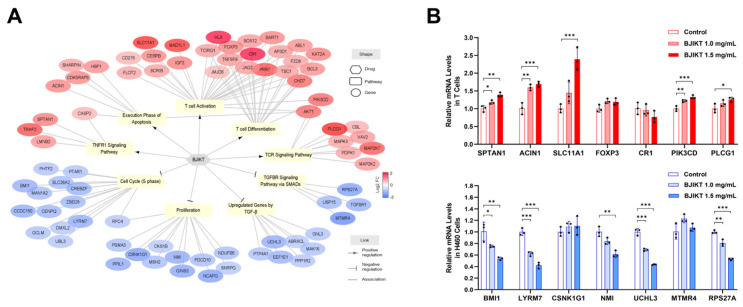
Drug–pathway–gene network analysis for the combination treatment of BJIKT and pembrolizumab. (**A**) The drug–pathway–gene network was reconstructed through transcriptome data analysis, illustrating the combinatorial effects of BJIKT and pembrolizumab. In this network, each element is distinctly represented: genes are depicted by circles, pathways by squares, and drugs by hexagons. Node color reflects the log_2_-transformed fold change (FC) of gene expression levels relative to pembrolizumab monotherapy. The links in the network indicate different types of relationships: an arrow end signifies activating (positive) interactions, a T-shaped end indicates inhibitory (negative) interactions, and a plain line denotes associations. Only significantly changed nodes (|FC| > 1.5, *p* < 0.05) within the pathway that consistently exhibited the same regulatory pattern as the pathway are shown. (**B**) Relative mRNA levels of specific genes in activated T cells and H460 cells following treatment with BJIKT. Values are presented as the mean ± SD (*n* = 3 per group). * *p* < 0.05, ** *p* < 0.01, and *** *p* < 0.001 compared between each group.

To clarify the molecular mechanisms underlying the combined effects of BJIKT and pembrolizumab on these pathways, we reconstructed a drug–pathway–Gene network (Figure 5A). BJIKT significantly downregulated 36 DEGs across the four inhibited pathways and upregulated 41 DEGs across the five activated pathways. This complex interaction indicates that the multi-component nature of BJIKT modulates multiple targets related to the cancer immune response in conjunction with pembrolizumab.

To support the observed molecular mechanisms of the combined effects of BJIKT and pembrolizumab, we conducted in vitro experiments using T cells and H460 cells treated with BJIKT. Activated T cells and H460 cells (5 × 10^5^ cells/well) were treated with BJIKT at concentrations of 1.0 mg/mL and 1.5 mg/mL for 24 h. As shown in Figure 5B, PCR analysis revealed significant changes in the expression of specific genes associated with the cancer immune response. In activated T cells, BJIKT treatment resulted in the increased expression of genes promoting T cell activation and TNF signaling, including SPTAN1, ACIN1, SLC11A1, PIK3CD, and PLCG1. Similarly, in H460 cells, treatment with BJIKT led to a downregulation of genes related to cell proliferation and TGF-β signaling, including BMI1, LYRM7, NMI, UCHL3, and RPS27A. These results suggest that BJIKT improves T cell function and TNF signaling while suppressing TGF-β signaling through multiple gene regulation, thereby enhancing the anticancer effects of pembrolizumab.

## 4. Discussion

This study demonstrated the profound immunomodulatory effects of combination therapy involving BJIKT and pembrolizumab in a preclinical model of human PBMC-injected H460 tumor-bearing MHC I/II dKO NSG mice. These findings underscore the ability of herbal medicines to synergize with the TME, shedding light on the intricate interplay and contributing to the observed anti-tumor effects. NSCLC is a primary contributor to cancer-associated deaths worldwide [18]. The TME is pivotal for the advancement, spread, and resistance to therapy in NSCLC [19,20]. Moreover, with their extensive history in traditional medical practices, herbal medicines have garnered recent interest as potential sources of new therapeutic agents targeting TME [21,22,23].

In a previous study [14], we identified the immune-mediated anti-tumor effects of a combined treatment comprising BJIKT and an anti-PD-1 antibody using a KLN205 tumor syngeneic mouse model, which is an immunocompetent model for studying the effects of immunotherapy. However, this model does not fully replicate the intricate interactions between the human immune system and TME. To address this limitation, we used humanized mouse models with human lung cancer immune systems to better reflect the human TME more accurately than the KLN205 mouse model. A humanized mouse model for NSCLC is best achieved using MHC I/II dKO NSG mice injected with H460 tumors and PBMCs. This choice was supported by two main reasons. First, these mice provide an immunodeficient setting that promotes successful engraftment and growth of human tumors, allowing the study of tumor biology and therapeutic responses with reduced rejection. Second, introducing human PBMCs into tumor-bearing mice replicates essential interactions between the tumor and human immune system, enabling the investigation of immune-mediated responses, including anti-tumor immunity and potential immunotherapies. This model creates a physiologically relevant microenvironment to study the immuno-oncological aspects of NSCLC.

The observed correlation between CD3, CD4, and CD8 T cells and NSCLC underscores the complex interaction between the TME and the immune system [24,25,26]. CD3+ T cells in the TME often indicate a sustained immune response [27]. In particular, the balance between CD4+ and CD8+ T cell subsets is vital in shaping the anti-tumor immune environment [28,29]. CD4+ T cells, known for their helper function, play a pivotal role in regulating immune responses, whereas CD8+ T cells are recognized for their cytotoxic activity against tumor cells [30,31,32]. As shown in Figure 3, the enhanced infiltration of immune cells, including CD3, CD4, and CD8 T cells, within the tumor tissues indicated an immunostimulatory response triggered by the combination treatment. This heightened immune cell presence is consistent with the observed reduction in exhaustion markers, such as LAG-3, TIGIT, and TIM-3, emphasizing the potential of BJIKT and pembrolizumab to mitigate T cell exhaustion and sustain a robust anti-tumor immune response. T cells with elevated exhaustion markers may exhibit reduced cytotoxic activity, thereby diminishing their ability to effectively target and eliminate cancer cells [33,34]. Collectively, these findings indicate multifaceted immunomodulation by combination therapy. The coordinated interplay between immune cell infiltration and exhaustion marker regulation and the subsequent impact on tumor growth highlight the potential of BJIKT and pembrolizumab as a synergistic therapeutic approach for NSCLC.

To investigate the combined effects of BJIKT and pembrolizumab, transcriptome analysis using RNA-seq data revealed a distinct gene expression profile for the combined therapy compared to pembrolizumab alone. This analysis identified 554 DEGs, with 301 upregulated and 253 downregulated genes. GSEA revealed the synergistic effects of BJIKT and pembrolizumab at the cellular pathway level. Specifically, combinatorial treatment with BJIKT enhanced T cell function by upregulating genes associated with T cell activation, differentiation, and the TCR signaling pathway. Additionally, this combined therapy activated the TNF signaling pathway while inhibiting the immunosuppressive TGF-β signaling pathway. Cancer cells can induce an immunosuppressive condition through the expression of cytokines, including TGF-β and vascular endothelial growth factor. Our study also showed that BJIKT inhibits the TGF-β signaling pathway. By examining cellular phenotype markers, we observed suppressed pathways related to the cell cycle and proliferation, coupled with enhanced apoptosis, following BJIKT treatment. NSCLC cells frequently exhibit resistance to apoptosis, allowing them to evade normal programmed cell death [35,36,37]. This resistance contributes to uncontrolled cell proliferation, survival, and tumor development [38,39,40]. In this study, we found that BJIKT potentially activates the anti-tumor immune response of pembrolizumab in NSCLC by modulating the mechanisms associated with apoptosis and cell proliferation.

To uncover the molecular mechanisms underlying these synergistic effects, we constructed a drug–pathway–gene network. BJIKT downregulated 36 DEGs in four inhibited pathways and upregulated 41 DEGs in five activated pathways, highlighting its multi-component nature. These interactions, which influence targets associated with the cancer immune response, suggest that BJIKT enhances T cell function and TNF signaling and suppresses TGF-β signaling, leading to cell cycle arrest, apoptosis, and amplification of pembrolizumab’s anticancer effects. Specifically, within the TNF signaling pathway, BJIKT notably increased the expression of SPTAN1, which may contribute to the modulation of apoptosis and inflammatory responses. In the execution phase of apoptosis, ACIN1 was significantly upregulated by BJIKT, reinforcing the role of BJIKT in promoting programmed cell death. Additionally, BJIKT treatment resulted in the increased expression of SLC11A1, a gene critical for T cell activation, indicating enhanced immune responsiveness. Moreover, key components of the TCR signaling pathway, PIK3CD and PLCG1, were upregulated by BJIKT, further supporting the enhanced activation of T cells. Furthermore, BJIKT treatment resulted in significant changes in gene expression in H460 cells. Specifically, BJIKT led to a notable decrease in the expression of BMI1 and LYRM7, both of which are involved in cell cycle regulation. Additionally, BJIKT reduced the expression of NMI, a gene associated with cancer cell proliferation. Furthermore, BJIKT treatment caused downregulation of UCHL3 and RPS27A, components of the TGF-β signaling pathway. These gene-level changes provide a deeper understanding of how BJIKT, in conjunction with pembrolizumab, orchestrates a multi-targeted approach to enhance anticancer efficacy.

Although these preclinical findings are promising, additional investigations are required to fully understand the precise molecular and cellular mechanisms that drive the observed effects. Specifically, while this study utilized humanized MHC I/II dKO NSG mice to model human tumor and immune system interactions, it is important to acknowledge the limitations of this model. Although the MHC I/II dKO NSG mice provide a relevant framework for studying human tumor biology and immune responses, they may not fully replicate the complexity of the human TME or systemic immune responses [41,42]. Consequently, the effects observed in these mice might not completely translate to human patients due to inherent differences between murine and human physiology. Translational studies involving human participants are crucial to validate the clinical relevance of these findings. Nevertheless, our study revealed the anti-tumor effects of the combined therapy comprising BJIKT and an anti-PD-1 antibody, mediated by the human immune system, focusing on the lung microenvironment. Validation studies of the transcriptome analysis results and further research on systemic immunity beyond the TME, such as the spleen, lymph nodes, and blood, are needed. Despite the need for further exploration, the presented data underscore the potential of combining BJIKT and pembrolizumab as a therapeutic strategy for NSCLC. Our study aimed to provide vital insights into the development of innovative treatment modalities that leverage the synergy between herbal medicine and an immune checkpoint blockade. This approach offers renewed hope to patients with NSCLC who remain refractory to conventional immunotherapeutic approaches.

## 5. Conclusions

Our findings suggest that the synergistic treatment altered immune cell populations and regulated the anti-tumor immune response, offering an additional understanding of BJIKT’s effects on the TME. This study showed that therapy with BJIKT and pembrolizumab suppressed tumor growth; increased tumor infiltration of CD3, CD4, and CD8; and decreased T cell exhaustion markers, such as LAG-3, TIGIT, and TIM-3, thereby demonstrating the potential to reverse T cell exhaustion in the lung microenvironment. Network analysis revealed that the combination therapy regulated various immune-related targets, such as enhancing T cell functions, activating the TNF signaling pathway, and inhibiting the immunosuppressive TGF-β signaling pathway. Therefore, a therapeutic approach integrating BJIKT and pembrolizumab holds promise as a practical strategy to augment the effectiveness of immunotherapy in patients diagnosed with NSCLC. Future research should focus on further elucidating the specific molecular mechanisms by which this combination therapy modulates the immune system, particularly in diverse NSCLC subtypes and different stages of disease progression. By understanding how this treatment modulates the immune response and reduces T cell exhaustion, researchers can continue to improve and optimize these approaches, ultimately providing new hope for patients to combat this type of lung cancer.

## Data Availability

The RNA-seq data generated in this study are available in the Gene Expression Omnibus (https://www.ncbi.nlm.nih.gov/geo/) under the accession code GSE260575.

## Abbreviations

BJIKT	Bojungikki-Tang
cDNA	Complementary DNA
DEGs	Differentially expressed genes
dKO	double knockout
FC	Fold-change
GSEA	Gene Set Enrichment Analysis
ICIs	Immune checkpoint inhibitors
IHC	Immunohistochemistry
IL	Interleukin
LAG-3	Lymphocyte activation gene-3
NES	Normalized enrichment scores
NSCLC	Non-small-cell lung cancer
PBMC	Peripheral blood mononuclear cells
PD-1	Programmed death 1
SD	Standard deviation
TGF-β	Transforming growth factor-β
TIGIT	T cell immunoreceptor with immunoglobulin and ITIM domain
TIM-3	T cell immunoglobulin and mucin domain-containing-3
TME	Tumor microenvironment
TNF	Tumor necrosis factor
TUNEL	Terminal deoxynucleotidyl transferase dUTP Nick End Labeling
